# Arterial Elastance: A Predictor of Hypotension Due to Anesthesia Induction

**DOI:** 10.3390/jcm12093155

**Published:** 2023-04-27

**Authors:** Serap Aktas Yildirim, Zeynep Tugce Sarikaya, Lerzan Dogan, Halim Ulugol, Bulent Gucyetmez, Fevzi Toraman

**Affiliations:** 1 Department of Anesthesiology and Reanimation, School of Medicine, Acibadem Mehmet Ali Aydinlar University, 34752 Istanbul, Turkey; 2 Department of Anesthesiology and Reanimation, Acibadem Altunizade Hospital, 34662 Istanbul, Turkey

**Keywords:** general anesthesia, post-induction hypotension, arterial elastance, ventriculoarterial coupling

## Abstract

Background: Hypotension is common after anesthesia induction and may have adverse outcomes. The aim of this study was to investigate whether arterial elastance (Ea) is a predictor of post-induction hypotension. Methods: Between January and June 2022, the hemodynamic parameters of 85 patients who underwent major surgery under general anesthesia were prospectively evaluated. The noncalibrated pulse contour device MostCare (Vytech, Vygon, Padua, Italy) was used to measure hemodynamic parameters before and after anesthesia induction. The duration of the measurements was determined from one minute before induction to 10 min after induction. Hypotension was defined as a greater than 30% decrease in mean arterial pressure from the pre-induction value and/or systolic arterial pressure of less than 90 mmHg. The patients were divided into post-induction hypotension (−) and (+) groups. For the likelihood of post-induction hypotension, a multivariate regression model was used by adding significantly different pre-induction parameters to the post-induction hypotension group. Results: The incidence of post-induction hypotension was 37.6%. The cut-off value of the pre-induction Ea for the prediction of post-induction hypotension was ≥1.08 mmHg m^−2^mL^−1^ (0.71 [0.59–0.82]). In the multivariate regression model, the likelihood of postinduction hypotension was 3.5-fold (1.4–9.1), increased by only an Ea ≥ 1.08 mmHg m^−2^mL^−1^. Conclusion: Pre-induction Ea showed excellent predictability of hypotension during anesthetic induction and identified patients at risk of general anesthesia induction-related hypotension.

## 1. Introduction

Hypotension is very common during and after anesthesia induction. A prolonged fasting period, a patient’s underlying comorbidities, a sympathetic blockade by anesthetic agents, vasodilation, a reduction in preload, and cardiac contractility can cause post-induction hypotension [1,2]. The relationship of even short-term hypotension with myocardial damage, renal injury, and stroke has been shown in many studies; therefore, it is very important to provide stable anesthesia induction [3]. In current anesthesia practice, we can only intervene when hypotension occurs. If we can identify patients who may experience hypotension during anesthesia induction before it occurs, we can prevent possible postoperative organ dysfunctions by reducing the duration and depth of hypotension with prophylactic fluid and vasopressor administration.

Previous studies have demonstrated that patients at risk of post-induction hypotension can be determined by variables such as the vena cava inferior collapsibility index, carotid intima-media thickness measurement, pulse pressure variation (PPV), and stroke volume variation (SVV) [4,5,6]. However, most studies evaluate the cardiovascular system with parameters that determine the preload and fluid responsiveness to predict the risk of post-induction hypotension, while the arterial system and its interaction with the ventricle are ignored [6,7,8]. The heart and the arterial system are both anatomically and functionally interconnected and do not act independently. The major determinant of cardiovascular system performance and cardiac energetics is ventriculoarterial coupling (VAC). Blood pressure is formed due to the interaction between the preload, the heart (contractility), and the arterial system [9]. The arterial load modulates cardiac function and maintains adequate perfusion pressure for the metabolic demands of organs. Therefore, blood pressure depends not only on the flow created by the heart but also on arterial load [10,11].

Ea is an indicator of cardiac afterload and arterial tone and can be calculated by the ratio of the end-systolic pressure (ESP) and stroke volume (SV) obtained from arterial wave analysis [11]. Ea provides information about the ESP/SV relationship and dynamic afterload and gives the clinician the opportunity to evaluate VAC indirectly. VAC can be calculated from the ratio of Ea to left ventricular elastane (Ees) using invasive methods as the gold standard [12].

The cardiovascular system maintains the VAC and provides adequate perfusion to the organs by adjusting the compliance and resistance of the arterial system according to the left ventricular performance in various physiological and pathological conditions [13].

If an adequate increase in left ventricle (LV) systolic functions is not achieved despite the increase in Ea, ventriculoarterial uncoupling occurs due to an Ea/Ees mismatch. Cardiovascular functional capacity may be reduced due to ventriculoarterial uncoupling in conditions such as aging, hypertension, heart failure, vascular stiffness, and elevated sympathetic tone [14].

We hypothesized that Ea values before anesthesia induction could predict post-induction hypotension. To test our hypothesis, we aimed to investigate the reliability of the Ea value, which was monitored preoperatively using the pressure analytical recording method (PRAM) to predict the risk of hypotension that may occur after anesthesia induction.

## 2. Materials and Methods

### 2.1. Ethical Approval

This prospective observational study was conducted between January and June 2022 at Acibadem Altunizade Hospital, which belongs to Acibadem MAA University. Ethical approval was obtained from the Regional Ethical Committee of Acibadem MAA University (ATADEK; 2021–10/21). The study complied with the Declaration of Helsinki on ethical principles for medical research involving human subjects. All patients gave informed consent before the study. Trial registration number NCT05648643.

### 2.2. Patients

Included in the study were patients with ASA physical status 1–3 who underwent elective major abdominal surgery under general anesthesia with intra-arterial blood pressure monitoring before induction. An inappropriate signal acquisition may occur due to some diseases and conditions (severe lung and valvular heart diseases) during the hemodynamic monitoring with the PRAM. Therefore, some patients were excluded from the study. Patients were excluded from the study if they were under 18 years of age and had arrhythmia (atrial fibrillation, frequent premature beat), severe pre-existing lung disease, severe valvular heart disease (aortic/mitral stenosis or insufficiency), congenital heart disease, chronic renal disease on dialysis, severe peripheral vascular disease, morbid obesity, or emergency surgery.

### 2.3. Study Protocol

Intravenous fluid and premedication were not administered to patients before induction. Solid food intake for at least eight hours and fluid intake for two hours were restricted before surgery. The monitoring method and timing were determined by the attending anesthesiologist for each patient. The patients scheduled for intra-arterial blood pressure monitoring prior to induction were included in the study. After arrival in the operating room, radial artery cannulation was performed with a 20-G catheter under local anesthesia, after which patients were asked to rest calmly for five minutes against the risk of tachycardia and hypertension due to anxiety before the pre-induction hemodynamic measurements. Hemodynamic variables, including systolic arterial pressure (SAP), mean arterial pressure (MAP), diastolic arterial pressure (DAP), heart rate (HR), stroke volume variation (SVV), pulse pressure variation (PPV), arterial elastance (Ea), cardiac power output (CPO), cardiac index (CI), and Dp/Dt were monitored using the uncalibrated pulse contour device MostCare (Vytech, Vygon, Padua, Italy), working with the PRAM. The duration of the measurements was defined from one minute before induction to 10 min after induction to ensure the detection of arterial hypotension associated with anesthesia induction without the effect of concomitant surgical stimulus and the patient positions. In all pre-induction measurements, patients were asked to take deep breaths six to eight times for one minute, and the average of the three recorded measurements was accepted as the baseline pre-induction value. Hemodynamic parameters recorded up to 10 min after induction were considered postinduction hemodynamic values. 

Each patient was administered intravenous Ringer’s lactate solution at a rate of 5 mL/kg, and general anesthesia was induced within one to two minutes with propofol (1.5–2.5 mg/kg), remifentanil (1 mcg/kg bolus), and rocuronium (0.6 mg/kg). After endotracheal intubation, mechanical ventilation was performed with a tidal volume of 8 mL/kg and a respiratory rate of eight to 12 breaths/min. General anesthesia was maintained with sevoflurane (1.5–2%) and remifentanil (0.1–0.2 mcg/kg/min). Hypnotics and opioids were titrated based on the patient’s hemodynamic response and bispectral index values (Covidien, Boulder, CO, USA). If the MAP was 30% below the preinduction value and/or the SAP was below 90 mmHg, hypotension was considered, and ephedrine was administered. The total administered ephedrine dose was recorded. Patients were classified into two groups according to the development of hypotension. Those who developed hypotension after induction were defined as the post-induction hypotension (+) group, and the remainder as the hypotension (−) group.

In the preoperative patient examination, the patient’s comorbidities were determined and recorded. Patients with a history of hypertension and/or using antihypertensive medication were considered to have hypertension. Patients using insulin and/or oral antidiabetic medication were considered to have diabetes mellitus. Patients were considered to have chronic renal failure if the markers indicating kidney damage were high for more than 3 months and/or the GFR was below 60 mL/min/1.73 m^2^. Patients with known reduced ejection fraction (<40%) were considered to have chronic heart failure.

In addition to hemodynamic parameters and comorbidities, patients’ ages, weights, heights, ASA scores, fasting times, whether they used beta-blockers and angiotensin-converting enzyme inhibitors, and whether mechanical bowel cleansing was performed were recorded.

### 2.4. Statistical Analysis

Descriptive data were presented as mean ± SD, median (quartiles), and percentages. The Kolmogorov–Smirnov test was used to detect normality. Student’s *t*-test, paired-student t, Mann–Whitney U, and Chi-square (Fisher’s Exact) tests were used to compare the groups. ROC curve analysis was used for significantly different parameters in the post-induction hypotension (+) group, and cut-off values were detected using Youden’s index. A multivariate logistic regression model was used to detect pre-induction predictors for the likelihood of post-induction hypotension. Pre-induction hemodynamic parameters that were significantly difference in hypotension group were added to the logistic regression model by using enter method. The primary outcome was to investigate whether Ea could predict post-induction hypotension. According to collected data at the end of the first 6 months, the estimated power for pre-induction Ea levels of groups was calculated as 0.99 (effect size: 1.2 for sizes 53 and 32, mean difference = 0.3 and ∝ = 0.05, GPower 3.1.9.4, Heinrich Heine University, Düsseldorf, Germany). SPSS Version 28 (IBM Corp., Armonk, NY, USA) was used for all the statistical analyses, and the *p*-value was accepted as <0.05 for significance.

## 3. Results

Eighty-five patients were included in the study. The demographic data of the patients are listed in Table 1. The most common comorbidity was hypertension (52.9%). In the pre-induction, Ea was significantly lower, whereas SAP, DAP, MAP, CI, CPO, Dp/Dt, SVV, and HR were significantly higher than those values in post-induction (*p* = 0.024, *p* < 0.001, *p* < 0.001, *p* < 0.001, *p* < 0.001, *p* < 0.001, *p* = 0.034, and *p* = 0.018, respectively). A comparison of the pre-induction and post-induction hemodynamic parameters of the patients is presented in Table 2.

Hypotension was observed in 32 patients (37.6%). Demographic data were similar between patients who developed hypotension and those who did not. The usage of laxatives, beta-blockers, ACE/AR medication, fasting hours, and administered IV fluid were similar between the hypotension (+) group and the hypotension (−) group. The SAP was similar between the hypotension (+) group and the hypotension (−) group before induction (147 ± 21 mm/Hg and 141 ± 24 mm/Hg, respectively; *p* = 0.196). In the post-induction hypotension (+) group, only pre-induction Ea and SVV were significantly higher than the post-induction hypotension (−) group (*p* < 0.001 and *p* = 0.036, respectively). After induction, there was a significant decrease in SAP, DAP, MAP, CI, and CPO Dp/Dt in the hypotension group (*p* < 0.001), while HR was similar in both groups (71 ± 12 bpm and 71 ± 14 bpm, *p* = 0.821). A comparison of the hypotension (+) group and the hypotension (−) group is presented in Table 3.

The cut-off values of Ea and SVV to detect post-induction hypotension were ≥1.08 mmHg m^−2^mL^−1^ (0.71 [0.59–0.82]) and ≥13% (0.62 [0.51–0.75]) (*p* = 0.002 and *p* = 0.049, respectively).

In the multivariate regression model, the likelihood of post-induction hypotension was 3.5-fold (1.4–9.1) increased by only pre-induction Ea ≥ 1.08 mmHg m^−2^mL^−1^ (*p* = 0.009; Table 4).

## 4. Discussion

To the best of our knowledge, this is the first study to evaluate the ability of preanesthetic Ea to predict hypotension after the induction of general anesthesia. The results of this study revealed that the pre-anesthetic Ea value was significantly associated with post-induction hypotension. The AUC and optimal cut-off value of pre-anesthetic Ea to predict hypotension were 0.71 and 1.08 mmHg m^−2^mL^−1^, respectively. Multivariate logistic regression analysis results showed that preanesthetic Ea over 1.08 mmHg m^−2^mL^−1^ is an independent risk factor for the incidence of postinduction hypotension.

Anesthesia induction-related hypotension is not uncommon and is still challenging for anesthesiologists to predict, despite advanced monitoring techniques. There are 140 different hypotension definitions identified in the literature. Frequently used hypotension definitions consist of an absolute decrease in systolic pressure or a relative decrease in systolic pressure or mean arterial pressure from baseline [3]. We used two of the most common definitions of hypotension in the present study. In current anesthesia practices, approximately 20–30% of patients develop hypotension during and after anesthesia induction [1]. In large observational studies, the relationship between intraoperative hypotension and adverse cardiac, renal, and cerebral outcomes has been reported [15,16]. Approximately one-third of intraoperative hypotension episodes occur during and after the anesthesia induction, and most can be prevented if foreseeable [17]. Therefore, we need quick and easy markers to predict patients at risk of hypotension to achieve the stable induction of anesthesia.

Many studies have used noninvasive and invasive monitoring techniques for the preoperative prediction of hypotension after anesthesia induction [4,5,7,18]. Anesthesia induction-related hypotension can be well predicted with preload variables such as pre-induction SVV, PPV, the Pleth variability index, and the ultrasonographic evaluation of major vascular structures such as the vena cava inferior and internal jugular vein measurement [5,6,7,18].

PPV and SVV were used to predict the patient volume status with cardiopulmonary interaction in mechanically ventilated individuals. However, studies show that these variables can provide reliable information about volume status by causing cardiopulmonary interaction with forced inspiration in spontaneous breathing, even in patients who are not mechanically ventilated.

Juri et al. reported that pre-anesthetic high SVV (during forced inspiratory breathing) is an independent risk factor in estimating hypotension and cardiac output decrease during anesthesia induction, with good sensitivity (83.9%) and specificity (78.6%). They found that the areas under the ROC curve (AUC) for preanesthetic SVV were 0.857 (95% confidence interval [CI], 0.747–0.967), with a cut-off value of 13%. Ali et al. reported that pre-induction PPV (> 16.5) during forced inspiratory breathing was a good predictor of post-induction hypotension (85% sensitivity and 90.5% specificity) [7].

In contrast, in the study of Jo et al., in which they aimed to identify patients at risk of hypotension associated with the beach chair position, they showed that pre-induction SVV in the spontaneous breathing state could not identify patients who developed hypotension, while the pre-induction stroke volume index (SVI; sensitivity 76.0%, specificity 60.0%) and cardiac index (CI; sensitivity 80.0%, specificity 66.7%) could [19]. They also demonstrated that cardiac performance is a more important determinant for identifying patients at risk for hypotension than preload variables and that patients with low SVI and CI are less able to tolerate changes in the preload. 

According to the present study, pre-anesthetic SVV (≥13%) during forced inspiratory breathing was not well correlated with hypotension that developed during anesthesia induction (OR = 1.5; *p* = 0.398). However, the inability of pre-induction forced inspiratory breathing maneuvers to create a constant tidal volume in each patient may have affected our results. Consequently, monitoring tidal volume as 8–10 mL per kilos during forced inspiration will give more reliable results. In addition, determining the risk of hypotension only through preload variables may not provide reliable results for every patient. A lack of good cardiovascular system performance to compensate for preload changes may indicate that the patient is more prone to hypotension.

The risk of hypotension is higher after anesthesia induction in patients with ASA scores above II, over 50 years of age, and with comorbidities (mostly hypertension) and a baseline MAP <70 mm/Hg [1,5]. In contrast, we found no correlations between post-induction hypotension and demographic variables, ASA status, fasting times, or pre-induction hemodynamic parameters. Our patient population consisted of patients with a high risk of post-induction hypotension. All patients were over 50 years old, and more than half had hypertension. Mostly, the ASA scores were II or above.

Demographic data, ASA scores, and comorbidities were similar between patients who developed hypotension and those who did not. There was no significant difference between factors affecting the patient’s volume status, such as fasting times, the amount of IV fluid given during induction, and the use of laxatives for bowel preparation.

Most studies have focused on the patient’s volume status and tried to determine the risk of hypotension with a hemodynamic index and measurements that determine preloads. However, previous clinical studies have proven that even prolonged fasting periods before surgery will not lead to volume depletion, and patients remain normovolemic [20]. Correspondingly, we think that cardiac performance plays a more important role in identifying patients at risk of hypotension during anesthesia induction rather than volume status.

In an experimental study by García et al., a strong relationship was found between LV ejection fraction (LVEF) and VAC, and it was illustrated that LVEF was mainly affected by changes in contractility and afterload rather than by changes in preload and heart rate [21,22].

In conditions such as hypertension, impaired ventricular function, aging, and increased sympathetic tone, the arterial load acting on the left ventricle may increase. Thus, Ea may increase [14,23]. Saba et al. reported that cardiac output was reduced and VAC was impaired in hypertensive patients with elevated Ea [24]. İn contrast, we found that pre-induction Ea was higher, but CI was similar in the hypotension (+) group compared to the hypotension (−) group (3.2 (2.8–4.1) vs. 3.1 (2.7–3.9) *p* = 0.267). After induction, Ea was similar in both groups, while CI was lower in the hypotension (+) group than in the hypotension (−) group (2.1 (1.9–2.5) vs. 2.5 (2.1–3.0), *p* < 0.001). There was no statistically significant difference in the pre-induction CI between the hypotension (+) and hypotension (−) groups. However, after induction, we observed that the CI decreased more in patients with higher pre-induction Ea. This illustrates that patients with high Ea are more prone to post-induction hypotension and that cardiac output decreases.

Maintaining the coupling of the LV ventricle and arterial system is essential for the efficient functioning of the cardiovascular system. Cardiovascular performance may be impaired under conditions in which Ea and Ees do not increase together. A high Ea at the end of the systole causes an increase in LV end-systolic volume and, thus, a decrease in LVSV.

The experimental study by Mannozzi et al. illustrated that with increased sympathetic tone during exercise in healthy canines, both Ea and Ees were increased together, and VAC was preserved [25]. However, after inducing heart failure, Ees could not increase, and VAC decreased despite the increase in Ea. Ea is often increased due to changes in the arterial system in elderly people and individuals with a comorbidity. In these individuals, the mismatch between Ea and Ees with increased sympathetic tone during exercise and anxiety may be even more pronounced.

We hypothesize that anesthesia induction-related vasodilation and a reduction in cardiac contractility may lead to impaired VAC, decreased cardiovascular performance, and postinduction hypotension in individuals with elevated Ea compared to individuals with low Ea.

Nishikawa et al. reported a decrease in Ees/Ea ratios after the induction of anesthesia with propofol in elderly patients [26]. They attributed this deterioration in VAC after induction to a decrease in myocardial contractility rather than to an increase in the afterload. In the current study, we posit that the decrease in myocardial contractility during induction may cause a further decrease in Ees/Ea ratios in patients with high pre-induction Ea and that the risk of post-induction hypotension is higher in these patients.

As Ea indirectly reflects VAC, bedside Ea could be a valuable index in anesthesia practice. One of the advantages of Ea is that it can be calculated without the need for complex, invasive monitoring. Current hemodynamic monitors or noninvasive echocardiography provide SV measurements. The end-systolic pressure used in the calculation of Ea can be accurately estimated by calculating 90% of the systolic pressure [11,13]. Thus, Ea can be easily calculated from the ESP/SV ratio at the bedside.

Knowing the pre-induction Ea will provide useful information for the clinician to plan for anesthesia induction (drug selection, dose setting, rate of administration, use, or preparation of vasoactive agents). The presence of this information will prevent unnecessary fluid loads used for the prophylaxis or treatment of perioperative hypotension.

The present study has some limitations. Hypotension during anesthesia induction may develop due to multifactorial causes. Estimating post-induction hypotension on only one hemodynamic parameter may not be appropriate for every patient. We also tried to determine the risk of hypotension by commenting on VAC (Ees/Ea) over Ea. However, we did not calculate Ees, which is one of the major parameters determining VAC, using echocardiography or an invasive approach. To determine whether VAC is impaired during induction in patients with high Ea, there is a need for studies in which left ventricular end-systolic elastance is calculated, in addition to the hemodynamic parameters used in the current study. Other limitations of our study are non-RCT design, single-center study, limited patient profile, and surgical intervention.

## 5. Conclusions

This study demonstrated that preanesthetic Ea can identify patients at risk for hypotension associated with anesthesia induction. The risk of hypotension is three times higher in patients with an Ea value above 1.08 mmHg m^−2^ mL^−1^.

## Figures and Tables

**Table 1:** Patients’ characteristics.

The number of the patients, *n* (%)	85 (100.0)
Age, years	63 (52–68)
Male, *n* (%)	63 (74.1)
BMI (kg/m^2^)	26.1 (23.5–29.3)
ASA score	2 (2–2)
Comorbidities, *n* (%)	
Hypertension	45 (52.9)
Diabetes mellitus	30 (35.3)
Coronary arterial disease	17 (20.0)
COPD	7 (8.2)
Cardiac failure	3 (3.5)
Cerebrovascular event	2 (2.4)
Chronic renal failure	2 (2.4)
Preoperative period	
The usage of beta-blockers	14 (16.5)
The usage of ACE/AR blockers	3 (3.5)
The usage of laxatives	50 (58.8)
Fasting, hours	10 (10–12)

ACE: angiotensin-converting enzyme; ASA: The American Society of Anesthesiologists; AR: angiotensin receptor; BMI: body mass index; COPD: chronic obstructive pulmonary disease.

**Table 2:** Comparisons of hemodynamic parameters between pre- and post-induction for all patients.

	Pre-Induction	Post-Induction	*p*
SAP (mmHg)	144 ± 22	107 ± 24	<0.001
DAP (mmHg)	72 (64–77)	56 (50–66)	<0.001
MAP (mmHg)	96 (86–105)	72 (62–85)	<0.001
Ea (mmHg m^−2^mL^−1^)	1.0 (0.8–1.1)	1.1 (0.9–1.3)	0.028
SVV (%)	13 ± 4	12 ± 5	0.034
PPV (%)	11 (9–14)	12 (9–17)	0.112
CI (L/min/m^2^)	3.2 (2.8–4.1)	2.4 (2.0–2.8)	<0.001
CPO (watt)	1.3 (1.1–1.8)	0.8 (0.6–1.0)	<0.001
Dp/Dt (mmHg/msn)	1.4 (1.1–1.8)	0.8 (0.6–1.0)	<0.001
HR (bpm)	72 (66–82)	69 (62–80)	0.018

SAP: systolic arterial pressure; DAP: diastolic arterial pressure; MAP: mean arterial blood pressure; Ea: arterial elastance; PPV: pulse pressure variation; SVV: stroke volume variation; HR: heart rate; CI: cardiac index; CPO: cardiac power output. Paired student-t and Wilcoxon signed-rank tests were used for comparisons.

**Table 3:** Comparison between post-induction hypotension (−) and (+) groups.

	Hypotension (−) (*n* = 53)	Hypotension (+) (*n* = 32)	*p*
Age, years	64 (53–68)	62 (51–68)	0.438
Male, *n* (%)	42 (79.2)	21 (65.6)	0.165
BMI (kg/m^2^)	26.1 (23.6–31.0)	25.3 (23.2–28.0)	0.425
ASA	2 (2–2)	2 (2–2)	0.118
**Comorbidities, *n* (%)**			
Hypertension	29 (54.7)	16 (50.0)	0.673
Diabetes mellitus	18 (34.0)	12 (37.5)	0.741
Coronary arterial disease	13 (24.5)	4 (12.5)	0.095
COPD	4 (7.5)	3 (9.4)	1
Cardiac failure	2 (3.8)	1 (3.1)	1
Cerebrovascular event	2 (3.8)	0 (0.0)	0.525
Chronic renal failure	2 (3.8)	0 (0.0)	0.525
**Preoperative period**			
The usage of beta-blockers	9 (17.0)	5 (15.6)	1
The usage of ACE/AR blockers	1 (0.0)	2 (6.3)	0.554
The usage of laxatives	32 (60.4)	18 (56.3)	0.708
Fasting, hours	10 (10–12)	12 (10–12)	0.075
Administered IV fluid, mL	150 (100–150)	150 (100–188)	0.596
**Pre-induction parameters**			
SAP (mmHg)	147 ± 21	141 ± 24	0.196
DAP (mmHg)	70 ± 9	71 ± 9	0.568
MAP (mmHg)	95 ± 12	94 ± 13	0.585
Ea (mmHg m^−2^mL^−1^)	0.9 ± 0.2	1.2 ± 0.3	<0.001
SVV (%)	12 ± 3	14 ± 5	0.036
PPV (%)	11 (9–14)	12 (10–14)	0.284
CI (L/min/m^2^)	3.2 (2.8–4.1)	3.1 (2.7–3.9)	0.267
CPO (watt)	1.4 (1.1–1.9)	1.2 (1.0–1.5)	0.078
Dp/Dt (mmHg/msn)	1.4 (1.2–1.9)	1.2 (1.0–1.7)	0.094
HR (bpm)	69 (66–79)	79 (67–85)	0.055
**Post-induction parameters**			
SAP (mmHg)	117 (108–134)	84 (75–90)	<0.001
DAP (mmHg)	63 ± 9	50 ± 5	<0.001
MAP (mmHg)	82 ± 12	61 ± 6	<0.001
Ea (mmHg m^−2^mL^−1^)	1.0 (0.9–1.3)	1.1 (0.9–1.2)	0.96
SVV (%)	11 ± 4	13 ± 5	0.148
PPV (%)	12 (8–15)	14 (10–20)	0.036
CI (L/min/m^2^)	2.5 (2.1–3.0)	2.1 (1.9–2.5)	<0.001
CPO (watt)	0.9 (0.8–1.1)	0.5 (0.5–0.7)	<0.001
Dp/Dt (mmHg/msn)	0.9 (0.8–1.1)	0.6 (0.5–0.8)	<0.001
HR (bpm)	71 ± 12	71 ± 14	0.821
Administered ephedrine, mg	0 (0–0)	10 (1–15)	<0.001

ACE: angiotensin converting enzyme; ASA: The American Society of Anesthesiologists; AR: angiotensin receptor; BMI: body mass index; COPD: chronic obstructive pulmonary disease; SAP: systolic arterial pressure; DAP: diastolic arterial pressure; MAP: mean arterial blood pressure; Ea: arterial elastance; PPV: pulse pressure variation; SVV: stroke volume variation; HR: heart rate; CI: cardiac index; CPO: cardiac power output.

**Table 4:** Logistic regression model for the likelihood of post-induction hypotension.

	OR (95% CI)	*p*
Ea ≥ 1.08 mmHg m^−2^mL^−1^	3.5 (1.4–9.1)	0.009
SVV ≥ %13	1.5 (0.6–4.0)	0.398

Ea: arterial elastance; CI: confidence interval; SVV: stroke volume variation; OR: odds ratio.

## Data Availability

The datasets for the current study are available from the corresponding author upon reasonable request.
